# Smoking patterns in Great Britain: the rise of cheap cigarette brands and roll your own (RYO) tobacco

**DOI:** 10.1093/pubmed/fdu048

**Published:** 2014-08-11

**Authors:** Anna B. Gilmore, Behrooz Tavakoly, Rosemary Hiscock, Gordon Taylor

**Affiliations:** Department for Health and UK Centre for Tobacco Control Studies, University of Bath, Bath, UK

**Keywords:** tobacco pricing, cheap cigarettes, RYO, Great Britain, inequalities

## Abstract

**Background:**

In Britain, the tobacco industry segments cigarettes into four price categories—premium, mid-price, economy and ultra-low-price (ULP). Our previous work shows that tobacco companies have kept ULP prices stable in real terms. Roll your own (RYO) tobacco remains cheaper still.

**Methods:**

Analysis of 2001–08 General Household Survey data to examine trends in use of these cheap products and, using logistic regression, the profile of users of these products.

**Results:**

Among smokers, the proportion using cheap products (economy, ULP and RYO combined) increased significantly in almost all age groups and geographic areas. Increases were most marked in under 24 year olds, 76% of whom smoked cheap cigarettes by 2008. All cheap products were more commonly used in lower socio-economic groups. Men and younger smokers were more likely to smoke RYO while women smoked economy brands. Smokers outside London and the South East of England were more likely to smoke some form of cheap tobacco even once socio-economic differences were accounted for.

**Conclusions:**

This paper demonstrates that cheap tobacco use is increasing among young and disadvantaged smokers compromising declines in population smoking prevalence. Thus, tobacco industry pricing appears to play a key role in explaining smoking patterns and inequalities in smoking.

## Background

Price increases are consistently reported as one of the most effective means of reducing tobacco consumption.^1^ In high-income countries a 10% increase in price generally leads to a 4% fall in consumption.^2,3^ The young and those of lower income and educational status are the most price sensitive.^3–6^

European Union (EU) legislation requires Member States to have a mixed tobacco excise structure, with a proportional (ad valorem) excise duty calculated on the maximum retail selling price, and a fixed (specific) excise duty calculated per unit of the product. During the study period a minimum overall excise duty of 57% of the retail selling price of the price segment most in demand, known as the most popular price category (MPPC), was required. In 2002 there was an additional requirement that the overall excise duty should not be <60 Euros per 1000 cigarettes (and 64 Euros from July 2006) for cigarettes in the MPPC.^7^ The UK has some of the highest tax rates in Europe, but since 2000 tax has only increased by the rate of inflation.^7^ RYO is currently taxed at half the level of manufactured cigarettes.^8^

Recent increases in the use of cheap tobacco including discount cigarettes and roll-your-own (RYO) tobacco has been observed in various countries including the UK,^9–12^ other EU member states,^13^ Canada, and the USA.^14–16^ Novel research from the UK has examined the role that tobacco industry pricing strategies may play in the growth of cheap cigarettes.^9^ This work shows that the industry differentially shifts tax increases between brand segments such that taxes on the cheapest ultra-low-price (ULP) brands are not always fully passed onto consumers while taxes on more expensive brands are consistently overshifted (i.e. the industry increases its price over and above the tax increase). Consequently, real prices of the cheapest cigarettes have remained largely unchanged since 2006 and the gap between the cheapest and most expensive cigarettes has widened.^9^ In the context of comprehensive marketing restrictions, the tobacco industry has also increasingly been using price-based marketing, including price-discounting and price-marking of cheap cigarette packs to promote its products.^17,18^ These findings raise concerns that tax and price policies may not be as effective as assumed and, in particular, may be least effective in those who smoke the cheapest cigarettes. These concerns are supported by recent evidence showing that the ready availability of cheap cigarettes constrains the ability of higher cigarette prices to promote smoking cessation.^19^

Smoking rates are higher and quit rates are lower, among the more deprived compared with the less deprived, in Scotland and Wales compared with England, and in the North compared with the South of England.^20–22^ In addition to higher smoking rates, the most disadvantaged start at a younger age, have higher rates of tobacco consumption and are less likely to quit.^20–22^ Recent reviews have concluded that price increases were the most likely intervention to reduce such inequalities.^20,23^

The evidence above highlights the importance of understanding the potential impacts of tobacco industry pricing strategies on patterns of tobacco use and whether they explain the smoking prevalence patterns outlined above. To explore this we need to understand trends in the use of cheap cigarettes and RYO as well as the profiles of users of such products. This paper aims to examine these issues and the attendant policy implications. In so doing it updates and extends previous work on this issue which explored use of so-called supermarket cigarettes brands in 1994.^24^ The market has changed considerably in the intervening period with the major cigarette companies buying up these supermarket brands and launching additional brands in this cheapest ULP segment.^7,17,25^

## Methods

### Data

The 2001–08 annual General Household Surveys (GHS) (in 2008 renamed the General Lifestyle Survey), designed to be representative of the population of Great Britain,^26^ were analysed. This study period was chosen because there were substantial changes in the GHS variables between 2000 and 2001 and GHS data sets were only available until 2008 at the time of analysis. The annual sample size in those aged 16 and over varied from 12 900 to 17 200, except in 2005 when the sample size increased to over 21 600 because the survey period changed from a financial year to a calendar year basis.^27^ The response rate ranged between 69 and 76% for these years.^28^ All analyses were weighted.^29^

We examined current adult smokers who gave full interviews: those aged 16 and over answering ‘yes’ to the question ‘do you smoke cigarettes at all nowadays?’ Current smokers were then asked ‘Do you mainly smoke filter-tipped cigarettes or plain or untipped cigarettes or hand-rolled cigarettes?’ Smokers of filter cigarettes were asked ‘Which brand of cigarette do you usually smoke?’ We allocated the brands named by respondents to one of four price segments—premium, economy, mid-price and ULP brands based on an extensive review of the literature combined with detailed brand-specific price data obtained from Retail Newsagent Price Checker (1999–2005) and AC Nielson (2006–9).^7,9^ The literature search, covering the period 1999–2009, identified market research reports, industry analyst reports, tobacco manufacturer annual reports, articles from the industry journal, *Tobacco Journal International*, and a single academic paper^24^ and was used to identify industry price segmentation and the brands sold within each price segment. Where brands could not be allocated to a particular price segment based on the literature, they were allocated on the basis of price. Smokers whose brand could not be allocated to a price segment [which occurred for 10% of brands (but only 12 respondents in the final analysis)] and cigarette smokers of plain tipped cigarettes were categorized as ‘other/did not answer’. For analysis, premium and mid-price brands were combined into an ‘expensive brands’ category as our prior work showed that the price of mid-price brands overlapped with the price of premium brands.^7^ Economy, ULP and RYO cigarettes, when referred to collectively, are called ‘cheap cigarettes’ and where just economy and ULP brands are referred to they are called ‘cheap brands’.

### Trends in smoking patterns

We first examined trends in the proportion of the Great Britain population smoking expensive (premium/mid-price), economy, ULP and RYO cigarettes. We then examined trends among cigarettes smokers overall and by age and location. Rates were presented graphically and 95% confidence intervals calculated using SPSS complex samples for 2008 where sample design information was available, and for the other years using 2008 defts.^30^

### Who smokes cheap tobacco?

Determinants of economy, ULP and RYO use, compared with expensive brand use, were explored through complex samples multinomial regression modelling. Analyses were restricted to the most recent year of data (2008). First a complex samples bivariate analysis was performed and then all variables were entered simultaneously with cigarette type as the outcome variable and gender, age group, ethnicity, National Statistics Socio-Economic Classification (NS-SEC), education and location as explanatory variables. We derived four age-group categories (16–24, 25–39, 40–54 and 55+years), two ethnicity categories (white British and other) and seven geographic areas (London; East and South East of England; West and East Midlands; North East, North West, Yorkshire and Humber; South West; Wales and Scotland). We used a five-category version of the NS-SEC: managerial and professional occupations, intermediate occupations, routine and manual occupations, never worked and long-term unemployed and full-time students. Educational qualifications were grouped into A-level or above, O-level or equivalent, basic or none and other/unknown. The model was checked for multicollinearity. All analyses were performed using SPSS versions 14 and 18.

## Results

### Trends in smoking patterns

#### Population prevalence trends

**Fig. 1:**
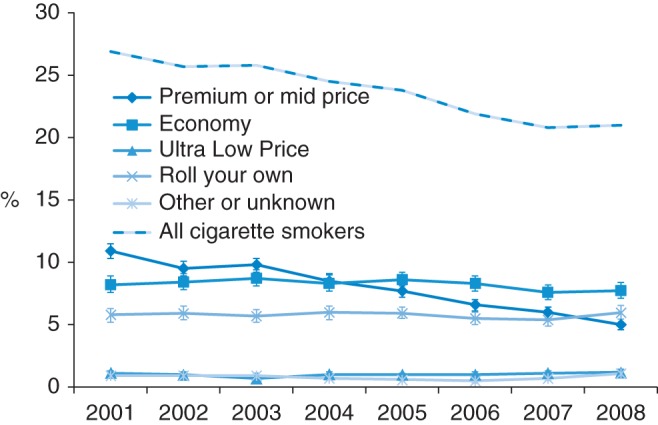
Smoking trends in the Great Britain population, 2001–08: overall smoking prevalence and the proportion of the Great Britain population smoking, expensive (premium and mid-price), economy, ULP and RYO cigarettes.

The marked decline in the smoking rate in the population as a whole over the period 2001–08, from 27 to 21%, reflects a reduction in the proportion smoking expensive brands (Fig. 1 and Supplementary data, Table A1). We do not know whether this reduction was the result of expensive brand smokers quitting, or down trading to cheaper brands or fewer smokers starting to smoke these brands. In contrast, the proportion of the population smoking cheap cigarettes has not changed significantly over this period; ∼8% smoked economy brands, 1% smoked ULP brands and 6% smoked RYO, although the percentage smoking ULP brands increased significantly from 0.7% in 2003 to 1.2% in 2008.

#### Trends among cigarette smokers

**Fig. 2:**
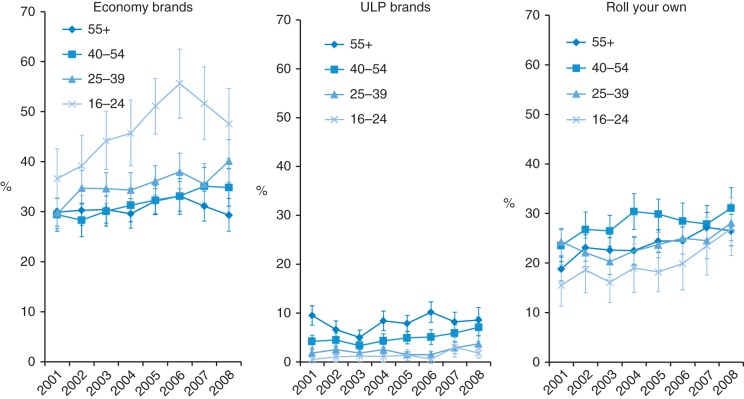
Trends in the proportion of cigarette smokers smoking economy, ULP and RYO cigarettes from 2001 to 2008 by age group (graphs show prevalence estimates and 95% confidence intervals).

By 2008 24% smokers smoked premium or mid-price brands, 37% smoked economy brands, 6% ULP brands and 28% smoked RYO with 5% unclassified (data not shown). Among cigarette smokers the proportion smoking cheap cigarettes (economy, ULP and RYO combined) increased significantly from 56% in 2001 to 71% in 2008 (Table 1). Cheap cigarette use increased significantly in all age groups except smokers aged over 55 years who had the highest level of cheap cigarette use in 2001. The most marked trends were seen in 16–24 year olds. This group was least likely to smoke cheap cigarettes in 2001 (53%) and most likely to do so in 2008 (76%); an absolute increase in cheap cigarette use of 24% compared with increases of 16, 16 and 6%, respectively, in each sequentially older age group. Among the cheap cigarette options, economy brand use was consistently highest in the youngest age group and increased significantly only in the two youngest groups (Fig. 2 and Supplementary data, Table A2). ULP use showed less clear trends over the period as a whole but increased significantly between the low prevalence point in 2003 and 2008 in the three older age groups and was most common in the oldest age group throughout the period studied.

**Table 1:** The proportion (95% CI) of smokers using cheap cigarettes^a^ in 2001 and 2008

	*2001 %*	*2008 %*
Total	56.1 (54.2–58.0)	71.0 (68.9–73.0)
Age		
55+	58.2 (55.2–61.2)	64.4 (60.9–67.7)
40–54	57.1 (53.7–60.5)	73.0 (69.5–76.2)
25–39	55.5 (52.2–58.8)	71.9 (67.9–75.5)
16–24	52.6 (46.6–58.7)	76.3 (70.0–81.6)
Location		
London	34.9 (28.4–41.5)	55.0 (46.7–63.1)
East and SE of England	56.4 (52.7–60.2)	65.5 (61.1–69.6)
West and East Midland	58.8 (54.2–63.3)	73.1 (68.0–77.6)
NE, NW, Yorkshire and Humber	57.7 (54.3–61.0)	72.9 (69.2–76.4)
South West	68.1 (62.8–73.5)	83.1 (77.8–87.3)
Wales	72.5 (66.5–78.6)	82.5 (75.6–87.8)
Scotland	56.1 (49.7–62.4)	74.1 (67.0–80.1)

^a^Cheap cigarettes include economy and ULP manufactured filter brands and RYO.

RYO use was higher in the intermediate age groups at the beginning of the decade, increased significantly in all age groups except 25–39 year olds (in whom economy and ULP brand use significantly increased) and by 2008 there were no significant age group differences (Fig. 2). The increase was most marked in the youngest, 16–24-year-old age group.

**Fig. 3:**
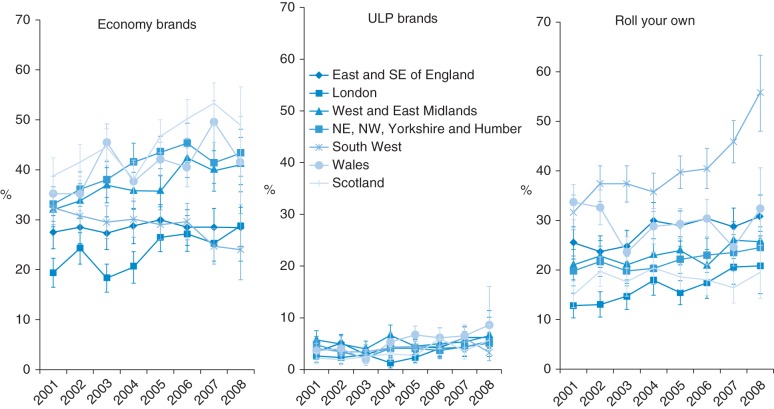
Trends in the proportion of cigarette smokers smoking economy, ULP and RYO cigarettes from 2001 to 2008 by location (graphs show prevalence estimates and 95% confidence intervals).

Cheap cigarette use significantly increased in all geographic areas except Wales which had the highest rates in 2001 (Table 1) and marked geographic inequalities were seen with rates in London significantly lower than in any other area (other than East of England in 2008). The specific form of cheap cigarette use and trends therein varied by area (Fig. 3 and Supplementary data, Table A3). Economy brand use was highest in Scotland throughout the period and grew significantly in Northern and Midland England, grew non-significantly in Scotland, Wales and London but remained steady in East and South East England and fell in the South West. The only location where ULP use grew significantly between 2001 and 2008 was Scotland. There was, however, a significant growth between the mid-2000s and 2008 in all locations except the South West and the Midlands.

RYO use grew non-significantly in most locations other than Wales, where no clear trend was seen, and the South West, where a marked and significant increase was seen. More than half of smokers in the South West now use RYO, significantly higher than in any other region.

### Who smokes cheap tobacco? (multinomial multivariable regression analysis)

There were 3172 current smokers in the 2008 General Lifestyle Survey. Two thousand and eight hundred and eighty-one of these completed full interviews and were aged over 16. Location or NS-SEC data were not available for 77 respondents who were therefore excluded from the analysis leaving 2804 cases (Table 2).

**Table 2:** Sample characteristics: proportion (and 95% confidence interval) of cigarette smokers smoking various types of cigarette by subgroup, 2008 data

		*Manufactured filter brands*			*% RYO*	*% other/DNA^b^*
			*Cheap manufactured filter brands*			
	**n*^a^*	*% premium/mid-price (*n* = 684)*	*% economy (*n* = 1004)*	*% ULP (*n* = 172)*	*(*n* = 794)*	*(*n* = 150)*
Gender						
Male	1306	23.0 (20.5–25.8)	29.5 (26.5–32.7)	4.6 (3.5–5.9)	37.8 (34.7–40.9)	5.1 (4.0–6.6)
Female	1498	25.0 (22.6–27.5)	43.7 (40.8–46.6)	6.7 (5.4–8.4)	19.7 (17.4–22.1)	4.9 (3.8–6.4)
Age						
55+	884	30.9 (27.7–34.3)	29.2 (26.1–32.6)	8.5 (6.5–11.1)	26.5 (23.5–29.8)	4.8 (3.5–6.7)
40–54	868	23.0 (19.9–26.4)	34.5 (30.8–38.4)	7.0 (5.3–9.2)	31.2 (27.4–35.3)	4.3 (3.0–6.1)
25–39	769	22.8 (19.5–26.5)	40.1 (35.9–44.4)	3.7 (2.6–5.3)	28.2 (24.6–32.0)	5.2 (3.6–7.4)
16–24	283	16.4 (11.4–23.1)	49.9 (42.5–57.3)	1.5 (0.5–4.1)	25.4 (19.7–32.0)	6.8 (4.2–10.8)
Ethnicity						
White British	2562	22.4 (20.6–24.3)	37.9 (35.5–40.4)	5.7 (4.7–6.8)	29.4 (27.2–31.7)	4.7 (3.9–5.7)
All other ethnicities	242	38.7 (31.4–46.5)	28.2 (21.8–35.8)	5.9 (3.1–11.0)	19.3 (14.0–26.0)	7.9 (4.4–13.8)
NS-SEC						
Managerial and professional occs	636	36.6 (32.6–40.7)	32.8 (28.7–37.1)	3.9 (2.6–5.8)	20.8 (17.4–24.7)	6.0 (4.2–8.5)
Intermediate occs	474	29.4 (25.0–34.2)	37.3 (32.2–42.7)	6.5 (4.4–9.5)	24.3 (19.9–29.3)	2.6 (1.6–4.2)
Routine and manual occs	1270	18.2 (15.8–20.9)	39.2 (35.8–42.6)	5.9 (4.7–7.4)	31.3 (28.2–34.6)	5.4 (4.1–7.1)
Never worked and LT unemployed	372	15.4 (11.5–20.1)	37.0 (31.6–42.7)	7.5 (5.0–11.0)	35.9 (30.7–41.6)	4.2 (2.5–7.1)
Full-time students	52	25.9 (9.9–52.7)	27.3 (15.7–43.1)	3.5 (0.9–13.4)	31.4 (18.1–48.7)	11.8 (4.6–27.3)
Education						
A level or more	827	33.4 (29.9–37.0)	32.4 (28.6–36.5)	4.5 (3.1–6.4)	24.6 (21.3–28.3)	5.1 (3.7–7.0)
O level or equivalent	752	21.1 (17.7–25.1)	40.9 (36.8–45.1)	5.6 (4.1–7.8)	27.8 (24.3–31.6)	4.5 (3.1–6.4)
Basic or none	987	17.8 (15.2–20.7)	39.6 (35.9–43.4)	7.3 (5.6–9.5)	31.5 (28.0–35.1)	3.8 (2.7–5.4)
Other or unknown	238	24.0 (18.3–30.9)	29.4 (23.1–36.6)	3.8 (1.9–7.2)	31.3 (24.6–38.8)	11.5 (7.2–18.0)
Location						
London	232	38.5 (30.8–46.9)	28.2 (21.1–36.5)	5.6 (2.6–11.7)	20.4 (15.1–27.0)	7.3 (4.4–12.0)
East and SE of England	604	29.9 (26.0–34.1)	29.1 (25.2–33.3)	6.5 (4.5–9.3)	30.5 (26.5–34.8)	4.0 (2.4–6.6)
West and East Midland	472	22.1 (17.9–27.0)	41.1 (35.7–46.8)	6.3 (4.0–9.8)	25.3 (20.8–30.5)	5.1 (3.3–7.8)
NE, NW, Yorkshire and Humber	781	21.6 (18.4–25.1)	43.5 (38.9–48.3)	5.0 (3.6–7.0)	24.3 (20.5–28.6)	5.6 (4.0–7.8)
South West	260	14.3 (10.7–18.8)	22.6 (17.2–29.2)	3.1 (1.6–5.9)	56.8 (49.2–64.1)	3.2 (1.5–6.9)
Wales	166	11.9 (8.1–17.1)	41.4 (32.8–50.6)	8.8 (4.5–16.4)	32.4 (25.0–40.8)	5.5 (2.4–11.9)
Scotland	289	21.5 (15.8–28.6)	48.4 (40.7–56.1)	5.6 (3.5–8.7)	19.8 (14.4–26.5)	4.7 (2.8–7.9)
Total	2804	24.1 (22.2–26.0)	36.9 (34.6–39.2)	5.7 (4.7–6.8)	28.3 (26.2–30.5)	5.0 (4.2–6.1)

^a^Unweighted.

^b^Other/DNA (did not answer) includes 24 cases where the type of cigarette smoked was unknown, 18 respondents who smoked plain or untipped cigarettes and 108 filter cigarette smokers of which 52 had no brand information available, 44 had no regular brand and 12 where the brand could not be allocated to a price category.

The multivariable analysis (Table 3) suggested that economy brands compared with expensive brands were more likely to be smoked by women rather than men, younger smokers, white British smokers rather than ethnic minorities, those in lower socio-economic and educational groups and in all locations (except the South West And East And South East of England) compared with London.

**Table 3:** Multinomial regression of three types of cheap cigarette use compared with expensive manufactured filter brands use (OR and 95% CI), 2008 data (*n* = 2804)

	*Bivariate OR (95% CI)*			*Multivariate OR (95% CI)*		
	*Cheap manufactured filter cigarettes*		*RYO*	*Cheap manufactured filter cigarettes*		*RYO*
	*Economy*	*ULP*		*Economy*	*ULP*	
Gender						
Male	1.00	1.00	1.00	1.00	1.00	1.00
Female	1.37 (1.10–1.70)	1.37 (0.96–1.95)	0.48 (0.38–0.60)	1.26 (1.01–1.58)	1.28 (0.89–1.85)	0.42 (0.34–0.53)
Age						
55+	1.00	1.00	1.00	1.00	1.00	1.00
40–54	1.59 (1.20–2.09)	1.10 (0.72–1.69)	1.58 (1.19–2.12)	2.12 (1.56–2.88)	1.38 (0.88–2.18)	2.12 (1.55–2.89)
25–39	1.85 (1.40–2.46)	0.59 (0.36–0.97)	1.44 (1.06–1.94)	3.00 (2.18–4.12)	0.86 (0.51–1.46)	2.33 (1.66–3.27)
16–24	3.21 (1.99–5.16)	0.33 (0.11–1.05)	1.80 (1.07–3.02)	4.77 (2.99–7.63)	0.37 (0.11–1.26)	2.34 (1.41–3.89)
Ethnicity						
White British	1.00	1.00	1.00	1.00	1.00	1.00
All other ethnicities	0.43 (0.29–0.65)	0.60 (0.29–1.27)	0.38 (0.25–0.59)	0.46 (0.31–0.67)	0.72 (0.33–1.57)	0.35 (0.21–0.59)
NS-SEC						
Managerial and professional occs	1.00	1.00	1.00	1.00	1.00	1.00
Intermediate occs	1.42 (1.02–1.97)	2.09 (1.11–3.93)	1.45 (1.00–2.12)	1.15 (0.80–1.64)	1.87 (0.97–3.61)	1.24 (0.83–1.86)
Routine and manual occs	2.40 (1.81–3.18)	3.07 (1.85–5.07)	3.02 (2.20–4.15)	1.68 (1.22–2.31)	2.43 (1.38–4.30)	2.41 (1.70–3.41)
Never worked and LT unemployed	2.69 (1.76–4.11)	4.61 (2.34–9.09)	4.12 (2.70–6.29)	1.96 (1.22–3.15)	3.66 (1.77–7.54)	3.26 (2.06–5.14)
Full-time students	1.18 (0.34–4.05)	1.28 (0.20–8.16)	2.13 (0.61–7.46)	0.53 (0.18–1.58)	2.66 (0.44–15.93)	**1.85 (0.63–5.42)**
Education						
A level or more	1.00	1.00	1.00	1.00	1.00	1.00
O level or equivalent	1.99 (1.45–2.73)	2.00 (1.14–3.49)	1.78 (1.30–2.46)	1.67 (1.20–2.33)	1.49 (0.85–2.61)	1.58 (1.13–2.21)
Basic or none	2.29 (1.73–3.01)	3.07 (1.90–4.98)	2.40 (1.78–3.22)	2.37 (1.71–3.29)	1.99 (1.15–3.45)	2.39 (1.68–3.38)
Other or unknown	1.26 (0.81–1.96)	1.17 (0.52–2.65)	1.77 (1.12–2.79)	1.49 (0.93–2.36)	0.89 (0.37–2.13)	1.87 (1.14–3.07)
Location						
London	1.00	1.00	1.00	1.00	1.00	1.00
East and SE of England	1.33 (0.82–2.15)	1.50 (0.59–3.85)	1.92 (1.19–3.09)	1.05 (0.66–1.69)	1.33 (0.54–3.30)	1.43 (0.83–2.45)
West and East Midlands	2.54 (1.51–4.28)	1.97 (0.72–5.35)	2.16 (1.29–3.59)	1.94 (1.16–3.25)	1.59 (0.59–4.30)	1.46 (0.84–2.54)
NE, NW, Yorkshire and Humber	2.76 (1.71–4.45)	1.60 (0.63–4.05)	2.12 (1.30–3.47)	1.86 (1.17–2.94)	1.23 (0.50–3.06)	1.31 (0.77–2.23)
South West of England	2.17 (1.21–3.89)	1.48 (0.48–4.54)	7.49 (4.31–13.03)	1.66 (0.94–2.94)	1.24 (0.41–3.76)	6.11 (3.28–11.39)
Wales	4.75 (2.44–9.25)	5.06 (1.65–15.50)	5.12 (2.77–9.47)	3.23 (1.67–6.25)	3.58 (1.16–11.01)	3.03 (1.56–5.89)
Scotland	3.08 (1.71–5.53)	1.79 (0.65–4.96)	1.73 (0.91–3.28)	2.66 (1.50–4.71)	1.45 (0.53–3.97)	1.27 (0.64–2.51)

*Note*: ‘Other/DNA’ (see Table 2) was included in the analysis but is not shown here.

There were fewer significant differences for ULP brands perhaps due to the small number of smokers in this category. ULP smokers, compared with smokers of expensive brands, were more likely to be in routine or manual occupations or be long-term unemployed, have basic or no educational qualifications and be living in Wales rather than London.

RYO smokers, compared with expensive cigarette smokers, were more likely to be men, younger smokers, white British, have routine or manual occupations or never worked or be long-term unemployed, have lower educational qualifications and be living in the South West of England or Wales compared with London.

We note that a dose–response relationship was seen between the likelihood of smoking cheap cigarettes (both economy and RYO) and declining age, lower social class and educational status and between smoking ULP cigarettes and lower social class. This persisted even once other variables had been controlled for.

## Discussion

### Main findings of this study

While overall rates of smoking in Great Britain have been falling, this decline was only seen in the proportion smoking expensive cigarettes; the proportion of the population smoking economy or RYO cigarettes has shown no significant change in the 2000s while there was a small rise in the proportion smoking ULP cigarettes between the middle and end of the study period.

Among smokers, the proportion smoking cheap tobacco products has increased in line with the growing market share of cheap cigarettes we document elsewhere.^9^ Consistent with evidence that the young are the most price sensitive,^3–6^ the increase in use of cheap cigarettes (economy, ULP and RYO combined and RYO in particular) is most marked in the youngest smokers, three quarters of whom now smoke cheap cigarettes. Conversely, the increase did not reach significance in two groups where cheap cigarette use was highest at the outset: Welsh smokers and smokers aged 55 and over.

Our results suggest that different population subgroups tend to select different cheap products. Men are more likely to smoke RYO while women smoke economy manufactured cigarettes. Welsh smokers had high rates of smoking all types of cheap cigarettes, smokers living in Scotland, the Midlands and Northern England were most likely to smoke cheap manufactured brands, whereas over 50% of smokers in the South West of England now use RYO. Cheap cigarette use is significantly higher outside the East/South East of England.

The locations where we observed the highest odds of smoking cheap cigarettes are those with the highest rates of smoking.^20–22^ For example, the odds of smoking all forms of cheap cigarette in Wales and economy brands in Scotland were about triple those in London and smoking rates in 2011 were 24% in both Scotland and Wales, compared with 19% in England.^31^ The odds of smoking both cheap manufactured brands and RYO were also highest in the most disadvantaged groups (whether measured by NS-SEC or education), with a clear dose–response relationship observed. As our data are cross-sectional, it is not possible to know the direction of causation: for instance whether disadvantaged smokers are more likely to choose cheap tobacco or whether cheap tobacco is more available in disadvantaged areas. However, our linked paper^9^ shows that tobacco companies have ensured the real price of their cheapest cigarettes remains almost static in real terms by absorbing the tax increases on these products rather than passing them onto smokers. This suggests that tobacco industry pricing and the availability of cheap tobacco may play a key part in determining these unequal smoking patterns; an issue that has been largely overlooked in debates on inequalities in smoking.

### What is already known

Despite significant changes in the market our findings are broadly consistent with earlier work by Jarvis^24^ which examined use of what was then supermarket cigarette brands (ULP brands before they had been acquired by the TTCs) but extends this work in exploring both trends over time and use of RYO. To our knowledge, this is the first time these patterns of tobacco use have been examined in detail in Britain and the work helps address the dearth of literature on industry pricing strategies and their impact.^32^

Jarvis found that use of supermarket (now ULP) brands was more common in disadvantaged smokers, older age groups and women,^24^ broadly consistent with our findings. The increase in use of cheap cigarettes is not unique to the UK and has, for example, been documented in other European Union member states,^13^ and North America.^14–16^ In line with our analysis, evidence from North America shows that use of cheap manufactured brands is associated with lower socio-economic status.^14–16^ Work in Australia shows that increases in the real price of budget brands led to reduced consumption of such brands along with reduced smoking prevalence in blue-collar workers.^33^

The marked growth of RYO in the South West but not in other areas suggests that cultural aspects may be important in smokers' choice of product, with both international and UK evidence suggesting that RYO users view RYO as less harmful.^34,35^ The South West of England is probably the most rural of the English regions.^36^ RYO use is also more common in rural areas of Malaysia, Thailand^37^ and New Zealand.^34^ Scotland also includes remote areas yet RYO use is particularly low. This may be because rural use is hidden by low use in large urban conurbations, largely absent in the South West of England.

### What this study adds

Studies of cessation interventions indicate that quit rates are lower among disadvantaged smokers, a pattern attributed to their lower success rate as evidence indicates that they attempt to quit at the same rate as more advantaged smokers.^21^ A number of reasons have been proposed for this including lack of support for quit attempts in part because those in their social network are more likely to smoke, greater addiction to tobacco, lower compliance with treatment and service failings.^21,38,39^ To date, the role that the availability and use of cheap, legal sources of tobacco could play has been largely overlooked. Our findings, along with well-established evidence on the importance of price in reducing tobacco use particularly in poorer smokers^3–6^ and newer evidence that the availability of cheap cigarettes reduces the ability of price to promote cessation^19^ and that use of cheap cigarettes reduces the likelihood of both cessation attempts and successful quitting^40^ suggest that this could play a key role in explaining the high smoking rates and low quit rates among the least well off. Moreover, addressing this pricing problem would be a far cheaper solution than the innovations in cessation service provision otherwise suggested.^39^

What is unique about this paper is that we examine different forms of cheap manufactured cigarette and RYO use in the same analysis. Generally, RYO use is contrasted with use of all manufactured cigarettes or all other tobacco.^37,41–43^ Only by monitoring different forms of cheap tobacco could we show that, although different sources of cheap tobacco were popular in different groups of smokers, use of cheap tobacco was widespread.

### Limitations

The analysis was based on a large, representative national survey which was unusual in collecting data on brand preference and was informed by a comprehensive review of industry and retail literature on brand segmentation and pricing.^9^ The GHS had a complex sample design, but cluster and strata design factors were not publically available until 2008 so the standard error of the smoking trends analysis was estimated using results from the 2008 data set.

Ideally, a cohort analysis would have been undertaken to examine whether an individual's brand preferences had changed over time and whether young smokers were making different choices to previous generations. This was impossible because the GHS pseudocohort (1972–2004) did not include brand data and despite GHS having a rolling cohort design from 2005, funding has not been available to release data to allow analysis on this basis.^44^

As only repeat cross-sectional data were available, we are unable to make causal attributions. For example, the flat rates of cheap cigarette and RYO use at population level could be due to smokers trading down from expensive cigarettes to cheaper versions, quit rates being lower in those smoking cheaper products (for example, if heavier, more addicted smokers tend to smoke cheaper brands), or those taking up smoking in recent years being more likely to smoke cheaper products. Most likely all three play a role. While the different profiles of those smoking economy, ULP and RYO cigarettes suggests that down-trading cannot be the sole reason, it is important to note that the decline in the prevalence of expensive cigarette use does not necessarily indicate that smokers of these cigarettes have quit. To some extent the underlying reason does not matter. Instead, what this study highlights is that the availability of cheap tobacco products and their differential selection by population subgroups with high rates of smoking is likely to be undermining efforts to reduce tobacco use and needs to be addressed.

### Implications for research and policy

In linked work, we show how the tobacco industry is using a sophisticated pricing strategy, keeping ULP very cheap, while increasing prices on more expensive brands and timing the price changes so as to exaggerate the price differentials when tobacco excise rates increase each year.^9^ This study shows how cheap tobacco use is growing, with the growth most marked in the young and rates of use highest in disadvantaged smokers, groups known to have the lowest quit rates.^21,45–47^

Increasing the price of the cheapest tobacco products so that the gap between the most expensive and cheapest products (whether manufactured cigarettes or RYO) is closed should help address this issue. This will require an increase in the specific element of tobacco excise^48^ and a ban on price promotions and below cost selling. The 2011 budget^49^ started the process of closing the price gap but in response Imperial Tobacco launched a new ‘make your own’ tobacco product—pre-made paper cylinders into which loose tobacco is inserted via a machine, a product sold at a discount to ULP brands.^50^ This indicates that a ban on product innovation may also be required, an issue explored elsewhere.^51^ Finally, the possibility of using price-cap regulation in the tobacco sector to address this issue^52^ would bring other benefits including an estimated £500 million in additional government revenue.^53^

Future research on quit rates needs to give far greater consideration to the role of price in cessation and research on inequalities in smoking needs to consider the impact of price differentials between the most expensive and cheapest products. Our findings also highlight the importance of routine surveys continuing to monitor patterns of smoking by product type and brand.

## Supplementary data

Supplementary data are available at *PUBMED* online.

## Funding

This work is funded by EC FP7 Grant Agreement HEALTH-F2-2009-223323, ‘Pricing Policies and Control of Tobacco in Europe (PPACTE)’. A.G. is supported by a Health Foundation Clinician Scientist Fellowship. A.G. and R.H. are members of the UK Centre for Tobacco Control Studies (UKCTCS), a UK Centre for Public Health Excellence. Funding to UKCTCS from the British Heart Foundation, Cancer Research UK, the Economic and Social Research Council, the Medical Research Council and the National Institute of Health Research, under the auspices of the UK Clinical Research Collaboration, is gratefully acknowledged. The funders played no role in the study design, analysis, and interpretation of data, nor writing of the report or the decision to submit the article for publication.

## Ethical approval

The study was approved by the University of Bath Research Ethics Approval Committee for Health.

## Authors’ contributions

A.G. conceived the idea for the study, obtained the funding, co-reviewed the background literature, supervised the analysis, wrote, edited and revised the paper. B.T. worked on the project full time and should also be considered as a first author; he compiled the data sets, obtained background literature and co-reviewed it, undertook initial analyses, and contributed to the editing of the paper. R.H. helped prepare the data set, undertook analyses, contributed to writing and editing of the paper. G.T. supervised the analysis, undertook some analyses and edited the paper.

## Supplementary Material

Supplementary Data
