# Supplementary material for: Smoking patterns in Great Britain: the rise of cheap cigarette brands and roll your own (RYO) tobacco
Source: J Public Health (Oxf). 2014 Aug 11;37(1):78–88. doi: 10.1093/pubmed/fdu048 (PMC4340325; doi:10.1093/pubmed/fdu048)
Supplement: Supplementary Data [file supp_37_1_78__index.html]

Smoking patterns in Great Britain: the rise of cheap cigarette brands and roll your own (RYO) tobacco — Supplementary Data 

# Smoking patterns in Great Britain: the rise of cheap cigarette brands and roll your own (RYO) tobacco

## Supplementary Data

Supplementary Data

**Files in this Data Supplement:**

- Supplementary Data - Docx file
