# Supplementary material for: Smoking patterns in Great Britain: the rise of cheap cigarette brands and roll your own (RYO) tobacco
Source: J Public Health (Oxf). 2014 Aug 11;37(1):78–88. doi: 10.1093/pubmed/fdu048 (PMC4340325; doi:10.1093/pubmed/fdu048)
Supplement: Supplementary Data [file supp_fdu048_fdu048supp.docx]

**Appendix (web only)**

**Appendix Table 1. Smoking trends in the Great Britain population, 2001 to 2008: population prevalence and (95% confidence interval)**

|  | 2001^1^  % | 2002^1^  % | 2003^1^  % | 2004^1^  % | 2005^1^  % | 2006^1^  % | 2007^1^  % | 2008^2^  % |
| --- | --- | --- | --- | --- | --- | --- | --- | --- |
|  | (N=15393) | (N=14827) | (N=17467) | (N=14926) | (N=21735) | (N=16603) | (N=15687) | (N=14730) |
| *Cigarette smokers* |  |  |  |  |  |  |  |  |
| Expensive | 10.9 (10.3 to 11.5) | 9.5 (8.9 to 10.1) | 9.8 (9.2 to 10.3) | 8.5 (8.0 to 9.1) | 7.7 (7.2 to 8.1) | 6.6 (6.1 to 7.0) | 6.0 (5.5 to 6.4) | 5.0 (4.6 to 5.5) |
| Economy | 8.2 (7.6 to 8.9) | 8.4 (7.8 to 9.1) | 8.7 (8.1 to 9.4) | 8.3 (7.7 to 9.0) | 8.6 (8.1 to 9.2) | 8.3 (7.7 to 8.9) | 7.6 (7.0 to 8.2) | 7.7 (7.1 to 8.4) |
| ULP | 1.1 (0.9 to 1.3) | 1.0 (0.8 to 1.2) | **0.7 (0.6 to 0.9)** | 1.0 (0.8 to 1.2) | 1.0 (0.8 to 1.1) | 1.0 (0.8 to 1.2) | 1.1 (0.9 to 1.3) | 1.2 (1.0 to 1.4) |
| Roll your own | 5.8 (5.2 to 6.3) | 5.9 (5.4 to 6.5) | 5.7 (5.2 to 6.2) | 6.0 (5.4 to 6.5) | 5.9 (5.5 to 6.4) | 5.5 (5.0 to 6.0) | 5.4 (4.9 to 5.9) | 6.0 (5.4 to 6.5) |
| Other/DNA^3^ | 0.9 (0.7 to 1.0) | 0.9 (0.7 to 1.1) | 0.9 (0.8 to 1.1) | 0.7 (0.5 to 0.8) | 0.6 (0.5 to 0.8) | 0.5 (0.4 to 0.7) | 0.7 (0.5 to 0.8) | 1.1 (0.9 to 1.3) |
|  |  |  |  |  |  |  |  |  |
| *Non cigarette smokers* | |  |  |  |  |  |  |  |
| Cigars/pipes^4^ | 1.8 (1.6 to 2.1) | 1.7 (1.5 to 1.9) | 1.3 (1.2 to 1.5) | 1.2 (1.0 to 1.4) | 1.1 (1.0 to 1.3) | 1.1 (0.9 to 1.2) | 0.9 (0.7 to 1.0) | 0.6 (0.5 to 0.8) |
| Non smoker^5^ | 71.3 (70.3 to 72.4) | 72.2 (71.2 to 73.3) | 72.5 (71.5 to 73.5) | 74.0 (73.0 to 75.0) | 74.7 (73.9 to 75.6) | 76.7 (75.8 to 77.6) | 77.9 (76.9 to 78.8) | 77.8 (76.8 to 78.8) |
|  |  |  |  |  |  |  |  |  |
| *Unknown*^6^ | 0.0 (0.0 to 0.0) | 0.3 (0.2 to 0.4) | 0.3 (0.2 to 0.4) | 0.2 (0.1 to 0.3) | 0.3 (0.2 to 0.4) | 0.3 (0.2 to 0.4) | 0.5 (0.3 to 0.6) | 0.6 (0.5 to 0.8) |
|  |  |  |  |  |  |  |  |  |
|  |  |  |  |  |  |  |  |  |
| All cheap cigarettes^7^ | 15.1 (14.2 to 15.9) | 15.3 (14.5 to 16.2) | 15.2 (14.4 to 16.0) | 15.3 (14.5 to 16.2) | 15.5 (14.8 to 16.2) | 14.8 (14.0 to 15.6) | 14.1 (13.3 to 15.0) | 14.9 (14.0 to 15.8) |
|  |  |  |  |  |  |  |  |  |

^1^Confidence intervals calculated from simple random sample standard error and deft calculated using SPSS complex samples for 2008 data

^2^Confidence intervals from SPSS complex samples

^3^Other/DNA(Did not answer) includes 24 cases where the type of cigarette smoked was unknown, 18 respondents who smoked plain or untipped cigarettes and 108 filter cigarette smokers of which 52 had no brand information available, 44 had no regular brand and 12 where the brand could not be allocated to a price category

^4^Smoked cigars more than once a month or pipes at all nowadays

^5^Includes 3 respondents who did not smoke cigarettes but whose cigar/pipe smoking status was unknown

^6^Respondents whose smoking status was unknown

^7^Economy, ULP and Roll your own combined

Green shading – significant decrease from 2001

Yellow shading – significant increase from 2001

Bold text indicates low ULP rates in the middle of the decade

**Appendix Table 2. Trends in the proportion (95% CI) of cigarette smokers smoking expensive, economy, ULP and RYO cigarettes from 2001 to 2008 by age-group**

|  | 2001^1^ | 2002^1^ | 2003^1^ | 2004^1^ | 2005^1^ | 2006^1^ | 2007^1^ | 2008^2^ |
| --- | --- | --- | --- | --- | --- | --- | --- | --- |
| *Age 55+* | (N=992) | (N=926) | (N=1062) | (N=898) | (N=1276) | (N=929) | (N=870) | (N=892) |
| Expensive | 37.0 (34.1 to 39.9) | 34.8 (31.8 to 37.8) | 37.2 (34.4 to 40.1) | 35.8 (32.8 to 38.9) | 33.1 (30.6 to 35.6) | 30.0 (27.1 to 32.9) | 29.1 (26.2 to 32.1) | 30.8 (27.6 to 34.2) |
| Economy | 29.9 (27.1 to 32.7) | 30.3 (27.3 to 33.2) | 30.4 (27.7 to 33.1) | 29.6 (26.7 to 32.6) | 32.1 (29.6 to 34.6) | 33.1 (30.1 to 36.0) | 31.1 (28.1 to 34.1) | 29.3 (26.1 to 32.6) |
| ULP | 9.5 (7.5 to 11.5) | 6.6 (4.9 to 8.4) | **5.0 (3.6 to 6.5)** | 8.4 (6.4 to 10.4) | 7.9 (6.3 to 9.5) | 10.2 (8.1 to 12.3) | 8.2 (6.2 to 10.2) | 8.6 (6.6 to 11.1) |
| Roll your own | 18.8 (16.4 to 21.2) | 23.1 (20.4 to 25.7) | 22.6 (20.1 to 25.1) | 22.5 (19.8 to 25.2) | 24.4 (22.1 to 26.7) | 24.5 (21.8 to 27.3) | 27.2 (24.3 to 30.2) | 26.5 (23.5 to 29.8) |
| Other/DNA | 4.8 (3.4 to 6.2) | 5.3 (3.8 to 6.7) | 4.7 (3.4 to 6.0) | 3.6 (2.4 to 4.9) | 2.5 (1.6 to 3.4) | 2.2 (1.2 to 3.2) | 4.3 (3.0 to 5.7) | 4.8 (3.4 to 6.7) |
|  |  |  |  |  |  |  |  |  |
| *40-54 years* | (N=1075) | (N=1025) | (N=1284) | (N=1039) | (N=1431) | (N=987) | (N=936) | (N=877) |
| Expensive | 40.9 (37.5 to 44.3) | 37.3 (33.9 to 40.8) | 37.2 (34.1 to 40.3) | 31.4 (28.2 to 34.7) | 29.9 (27.1 to 32.6) | 31.1 (27.8 to 34.5) | 28.0 (24.7 to 31.4) | 22.8 (19.7 to 26.2) |
| Economy | 29.4 (26.1 to 32.7) | 28.3 (25.0 to 31.7) | 30.1 (27.1 to 33.1) | 31.3 (28.0 to 34.7) | 32.3 (29.4 to 35.2) | 33.1 (29.5 to 36.6) | 35.1 (31.5 to 38.8) | 34.8 (31.1 to 38.6) |
| ULP | 4.2 (2.8 to 5.5) | 4.5 (3.1 to 5.9) | **3.3 (2.2 to 4.4)** | 4.3 (3.0 to 5.7) | 4.9 (3.7 to 6.2) | 5.1 (3.6 to 6.6) | 5.9 (4.2 to 7.6) | 7.1 (5.4 to 9.2) |
| Roll your own | 23.5 (20.3 to 26.8) | 26.8 (23.3 to 30.3) | 26.5 (23.4 to 29.6) | 30.4 (26.8 to 34.0) | 29.9 (26.8 to 32.9) | 28.5 (24.9 to 32.1) | 27.9 (24.2 to 31.6) | 31.1 (27.3 to 35.2) |
| Other/DNA | 2.0 (1.0 to 2.9) | 3.1 (1.8 to 4.2) | 3.0 (1.8 to 4.0) | 2.5 (1.3 to 3.6) | 3.0 (2.0 to 4.1) | 2.2 (1.1 to 3.3) | 3.1 (1.8 to 4.3) | 4.2 (2.9 to 6.0) |
|  |  |  |  |  |  |  |  |  |
| *25-39 years* | (N=1399) | (N=1236) | (N=1450) | (N=1159) | (N=1599) | (N=1113) | (N=867) | (N=788) |
| Expensive | 41.7 (38.6 to 44.9) | 38.0 (34.6 to 41.3) | 40.0 (36.9 to 43.1) | 39.0 (35.6 to 42.5) | 36.8 (33.9 to 39.8) | 32.5 (29.1 to 35.9) | 34.3 (30.3 to 38.2) | 22.9 (19.6 to 26.5) |
| Economy | 29.5 (26.6 to 32.6) | 34.7 (31.5 to 38.2) | 34.6 (31.6 to 37.8) | 34.3 (31.0 to 37.8) | 36.1 (33.3 to 39.2) | 38.0 (34.6 to 41.7) | 35.5 (31.7 to 39.6) | 40.1 (35.9 to 44.4) |
| ULP | 1.8 (1.1 to 2.5) | 2.5 (1.6 to 3.4) | **1.8 (1.1 to 2.5)** | 2.5 (1.6 to 3.5) | 1.5 (0.9 to 2.1) | 1.5 (0.8 to 2.2) | 2.9 (1.7 to 4.0) | 3.7 (2.6 to 5.3) |
| Roll your own | 24.3 (21.5 to 27.0) | 22.1 (19.3 to 25.0) | 20.3 (17.7 to 22.9) | 22.4 (19.5 to 25.4) | 23.7 (21.1 to 26.2) | 25.0 (21.8 to 28.1) | 24.5 (20.9 to 28.0) | 28.1 (24.5 to 31.9) |
| Other/DNA | 2.8 (1.7 to 3.7) | 2.6 (1.5 to 3.6) | 3.3 (2.2 to 4.4) | 1.7 (0.8 to 2.6) | 1.9 (1.1 to 2.6) | 3.1 (1.8 to 4.3) | 2.9 (1.5 to 4.2) | 5.3 (3.7 to 7.5) |
|  |  |  |  |  |  |  |  |  |
| *16-24years* | (N=547) | (N=536) | (N=602) | (N=483) | (N=681) | (N=428) | (N=396) | (N=324) |
| Expensive | 43.4 (37.1 to 49.8) | 37.0 (30.8 to 43.2) | 34.5 (28.7 to 40.3) | 29.8 (23.6 to 36.0) | 25.2 (20.3 to 30.2) | 22.0 (16.0 to 28.0) | 19.3 (13.3 to 25.2) | 16.6 (11.9 to 22.8) |
| Economy | 36.6 (30.7 to 42.6) | 39.2 (33.2 to 45.3) | 44.2 (38.4 to 50.1) | 45.7 (39.2 to 52.3) | 51.1 (45.5 to 56.6) | 55.6 (48.7 to 62.5) | 51.6 (44.4 to 58.9) | 47.6 (40.7 to 54.6) |
| ULP | 0.5 (-0.2 to 1.2) | 1.0 (0.0 to 2.1) | 1.2 (0.1 to 2.2) | 1.1 (0.0 to 2.3) | 1.4 (0.3 to 2.5) | 0.6 (-0.3 to 1.5) | 3.0 (1.0 to 5.0) | 1.8 (0.8 to 4.2) |
| Roll your own | 15.5 (11.3 to 19.7) | 18.6 (14.0 to 23.2) | 16.1 (12.0 to 20.2) | 19.0 (14.1 to 23.8) | 18.2 (14.2 to 22.3) | 19.9 (14.6 to 25.1) | 23.5 (17.6 to 29.3) | 26.9 (21.5 to 33.2) |
| Other/DNA | 3.9 (1.9 to 5.9) | 4.1 (2.0 to 6.2) | 4.0 (2.1 to 6.0) | 4.4 (2.1 to 6.6) | 4.1 (2.2 to 5.9) | 1.9 (0.3 to 3.5) | 2.7 (0.7 to 4.7) | 7.0 (4.6 to 10.7) |

^1^Confidence intervals calculated from simple random sample standard error and deft calculated using SPSS complex samples for 2008 data

^2^Confidence intervals from SPSS complex samples

Green shading – significant decrease from 2001

Yellow shading – significant increase from 2001

Bold text indicates low ULP rates in the middle of the decade

**Appendix Table 3. Trends in the proportion (95% CI) of cigarette smokers smoking expensive, economy, ULP and RYO cigarettes from 2001 to 2008 by location**

|  | 2001^1^ | 2002^1^ | 2003^1^ | 2004^1^ | 2005^1^ | 2006^1^ | 2007^1^ | 2008^2^ |
| --- | --- | --- | --- | --- | --- | --- | --- | --- |
| *East & SE England* | (N=895) | (N=912) | (N=898) | (N=795) | (N=1128) | (N=762) | (N=701) | (N=631) |
| Expensive | 40.9 (37.3 to 44.6) | 38.8 (35.2 to 42.3) | 41.7 (38.1 to 45.3) | 34.3 (30.6 to 38.0) | 34.5 (31.4 to 37.7) | 34.1 (30.3 to 37.9) | 34.0 (30.1 to 38.0) | 30.1 (26.3 to 34.3) |
| Economy | 27.5 (24.2 to 30.8) | 28.5 (25.2 to 31.8) | 27.3 (24.0 to 30.6) | 28.8 (25.2 to 32.3) | 30.0 (27.0 to 33.0) | 28.5 (24.9 to 32.0) | 28.5 (24.8 to 32.3) | 28.4 (24.7 to 32.5) |
| ULP | 3.4 (2.0 to 4.9) | 5.1 (3.5 to 6.8) | **2.9 (1.6 to 4.3)** | 4.3 (2.7 to 6.0) | 4.4 (3.0 to 5.8) | 4.2 (2.5 to 5.9) | 6.2 (4.1 to 8.3) | 6.2 (4.3 to 8.9) |
| Roll your own | 25.5 (22.2 to 28.7) | 23.7 (20.5 to 26.9) | 24.8 (21.6 to 28.0) | 29.9 (26.3 to 33.6) | 28.9 (25.8 to 31.9) | 30.4 (26.7 to 34.2) | 28.7 (24.8 to 32.5) | 30.8 (26.9 to 35.1) |
| Other/DNA | 2.6 (1.3 to 4.0) | 3.9 (2.3 to 5.5) | 3.3 (1.8 to 4.8) | 2.7 (1.3 to 4.1) | 2.2 (1.1 to 3.3) | 2.8 (1.3 to 4.3) | 2.6 (1.1 to 4.1) | 4.4 (2.8 to 7.0) |
| *London* | (N=438) | (N=391) | (N=406) | (N=333) | (N=473) | (N=304) | (N=246) | (N=237) |
| Expensive | 61.8 (58.2 to 65.4) | 55.6 (52.0 to 59.2) | 60.7 (57.1 to 64.3) | 57.8 (54.0 to 61.7) | 52.5 (49.2 to 55.8) | 48.0 (44.0 to 51.9) | 45.5 (41.3 to 49.6) | 37.6 (30.0 to 45.8) |
| Economy | 19.4 (16.5 to 22.3) | 24.3 (21.1 to 27.4) | 18.3 (15.5 to 21.1) | 20.5 (17.3 to 23.6) | 26.5 (23.6 to 29.4) | 27.2 (23.7 to 30.8) | 25.3 (21.7 to 28.9) | 28.8 (21.7 to 37.0) |
| ULP | 2.6 (1.4 to 3.8) | 2.3 (1.1 to 3.4) | 2.9 (1.6 to 4.2) | **1.3 (0.3 to 2.2)** | 2.3 (1.3 to 3.3) | 4.1 (2.4 to 5.8) | 4.3 (2.5 to 6.1) | 5.5 (2.5 to 11.4) |
| Roll your own | 12.8 (10.3 to 15.3) | 13.0 (10.5 to 15.6) | 14.7 (12.0 to 17.3) | 17.9 (14.9 to 21.0) | 15.4 (13.0 to 17.8) | 17.4 (14.3 to 20.5) | 20.6 (17.1 to 24.0) | 20.8 (15.2 to 27.8) |
| Other/DNA | 3.4 (1.9 to 4.9) | 4.8 (3.0 to 6.6) | 3.4 (1.9 to 4.9) | 2.5 (1.1 to 3.9) | 3.3 (1.9 to 4.6) | 3.3 (1.7 to 4.9) | 4.3 (2.4 to 6.3) | 7.4 (4.4 to 12.1) |
| *West & East Mids* | (N=640) | (N=540) | (N=837) | (N=618) | (N=806) | (N=568) | (N=524) | (N=480) |
| Expensive | 39.0 (35.4 to 42.6) | 35.4 (32.0 to 38.9) | 33.2 (29.7 to 36.7) | 31.2 (27.6 to 34.8) | 32.3 (29.2 to 35.4) | 28.5 (24.9 to 32.1) | 25.6 (21.9 to 29.2) | 21.7 (17.6 to 26.5) |
| Economy | 32.0 (28.6 to 35.4) | 33.8 (30.3 to 37.2) | 36.9 (33.4 to 40.4) | 35.8 (32.1 to 39.5) | 35.7 (32.6 to 38.8) | 42.4 (38.5 to 46.3) | 39.8 (35.7 to 43.8) | 41.0 (35.6 to 46.5) |
| ULP | 5.7 (3.9 to 7.5) | 4.9 (3.2 to 6.5) | 4.0 (2.5 to 5.5) | 6.6 (4.6 to 8.6) | 4.5 (3.0 to 5.9) | 5.0 (3.2 to 6.8) | 5.3 (3.4 to 7.3) | 6.5 (4.1 to 10.1) |
| Roll your own | 21.0 (18.0 to 24.1) | 22.8 (19.6 to 25.9) | 21.0 (18.0 to 24.1) | 22.9 (19.6 to 26.3) | 23.9 (21.0 to 26.7) | 20.8 (17.5 to 24.1) | 26.0 (22.3 to 29.7) | 25.7 (21.1 to 30.9) |
| Other/DNA | 2.2 (1.0 to 3.5) | 3.2 (1.7 to 4.6) | 4.9 (3.1 to 6.7) | 3.5 (1.8 to 5.1) | 3.7 (2.3 to 5.1) | 3.3 (1.7 to 4.9) | 3.3 (1.6 to 5.0) | 5.2 (3.4 to 7.9) |
| *NE, NW, Y&H* | (N=1113) | (N=1005) | (N=1155) | (N=1024) | (N=1339) | (N=954) | (N=821) | (N=797) |
| Expensive | 38.3 (34.8 to 41.9) | 35.9 (32.4 to 39.4) | 36.0 (32.4 to 39.5) | 31.8 (28.2 to 35.4) | 27.9 (25.0 to 30.8) | 25.7 (22.2 to 29.2) | 26.8 (23.1 to 30.4) | 21.5 (18.4 to 24.9) |
| Economy | 33.1 (29.7 to 36.6) | 36.1 (32.7 to 39.6) | 38.0 (34.5 to 41.6) | 41.5 (37.6 to 45.3) | 43.5 (40.2 to 46.7) | 45.3 (41.4 to 49.3) | 41.4 (37.3 to 45.5) | 43.3 (38.7 to 48.1) |
| ULP | 4.8 (3.1 to 6.4) | 3.4 (2.0 to 4.8) | **2.3 (1.2 to 3.5)** | 4.1 (2.4 to 5.7) | 4.1 (2.8 to 5.5) | 3.7 (2.1 to 5.3) | 4.4 (2.6 to 6.2) | 5.1 (3.7 to 7.1) |
| Roll your own | 19.8 (16.8 to 22.8) | 21.7 (18.7 to 24.8) | 19.8 (16.8 to 22.8) | 20.3 (17.1 to 23.5) | 22.1 (19.3 to 24.8) | 23.0 (19.5 to 26.4) | 23.5 (19.9 to 27.1) | 24.5 (20.7 to 28.7) |
| Other/DNA | 4.0 (2.3 to 5.6) | 2.8 (1.4 to 4.1) | 3.9 (2.3 to 5.6) | 2.4 (1.0 to 3.7) | 2.4 (1.3 to 3.6) | 2.3 (0.9 to 3.7) | 3.9 (2.1 to 5.8) | 5.6 (4.0 to 7.8) |
| *South West* | (N=324) | (N=329) | (N=409) | (N=290) | (N=481) | (N=336) | (N=271) | (N=268) |
| Expensive | 28.9 (25.6 to 32.2) | 24.9 (21.8 to 28.1) | 26.1 (22.9 to 29.4) | 26.2 (22.8 to 29.7) | 25.1 (22.2 to 27.9) | 23.6 (20.2 to 27.0) | 21.6 (18.2 to 25.1) | 13.8 (10.3 to 18.2) |
| Economy | 32.4 (29.0 to 35.9) | 30.8 (27.5 to 34.2) | 29.5 (26.2 to 32.8) | 30.1 (26.5 to 33.6) | 29.0 (26.1 to 32.0) | 29.6 (26.0 to 33.3) | 24.7 (21.1 to 28.3) | 24.0 (18.0 to 31.2) |
| ULP | 4.1 (2.6 to 5.7) | 3.4 (2.0 to 4.8) | 3.4 (2.0 to 4.8) | 4.2 (2.5 to 5.8) | 4.4 (3.0 to 5.9) | 4.5 (2.7 to 6.2) | 6.1 (4.0 to 8.2) | 3.3 (1.7 to 6.0) |
| Roll your own | 31.6 (28.1 to 35.1) | 37.4 (33.8 to 41.0) | 37.4 (33.7 to 41.0) | 35.7 (31.8 to 39.5) | 39.7 (36.4 to 43.0) | 40.4 (36.4 to 44.5) | 45.9 (41.6 to 50.1) | 55.8 (48.0 to 63.3) |
| Other/DNA | 2.9 (1.5 to 4.4) | 3.4 (1.9 to 4.9) | 3.7 (2.1 to 5.2) | 3.8 (2.1 to 5.6) | 1.8 (0.8 to 2.8) | 1.9 (0.7 to 3.2) | 1.7 (0.5 to 3.0) | 3.1 (1.4 to 6.6) |
| *Wales* | (N=204) | (N=201) | (N=252) | (N=167) | (N=243) | (N=171) | (N=173) | (N=169) |
| Expensive | 23.8 (20.7 to 27.0) | 20.7 (17.8 to 23.7) | 24.4 (21.3 to 27.6) | 25.3 (21.9 to 28.7) | 18.7 (16.1 to 21.2) | 22.3 (19.0 to 25.6) | 17.3 (14.1 to 20.4) | 12.1 (8.2 to 17.6) |
| Economy | 35.2 (31.7 to 38.7) | 35.2 (31.8 to 38.7) | 45.5 (41.9 to 49.2) | 37.6 (33.9 to 41.4) | 42.2 (39.0 to 45.5) | 40.5 (36.6 to 44.4) | 49.6 (45.5 to 53.8) | 41.6 (33.0 to 50.7) |
| ULP | 3.6 (2.2 to 5.1) | 4.1 (2.6 to 5.7) | **1.9 (0.8 to 2.9)** | 5.3 (3.5 to 7.1) | 6.7 (4.9 to 8.4) | 6.1 (4.1 to 8.1) | 6.5 (4.3 to 8.6) | 8.6 (4.4 to 16.1) |
| Roll your own | 33.7 (30.1 to 37.2) | 32.6 (29.1 to 36.1) | 23.5 (20.3 to 26.7) | 28.8 (25.2 to 32.4) | 29.3 (26.3 to 32.4) | 30.4 (26.7 to 34.2) | 24.5 (20.8 to 28.1) | 32.3 (24.9 to 40.6) |
| Other/DNA | 3.6 (2.1 to 5.2) | 7.3 (5.1 to 9.4) | 4.7 (2.9 to 6.5) | 2.9 (1.4 to 4.4) | 3.1 (1.8 to 4.4) | 0.7 (-0.1 to 1.4) | 2.2 (0.8 to 3.5) | 5.4 (2.4 to 11.8) |
| *Scotland* | (N=399) | (N=345) | (N=441) | (N=352) | (N=517) | (N=362) | (N=333) | (N=297) |
| Expensive | 40.5 (36.9 to 44.2) | 34.2 (30.7 to 37.6) | 34.2 (30.7 to 37.7) | 35.7 (31.9 to 39.4) | 29.3 (26.4 to 32.3) | 24.3 (20.9 to 27.7) | 23.4 (19.8 to 26.9) | 21.3 (15.7 to 28.2) |
| Economy | 38.8 (35.3 to 42.4) | 41.5 (37.9 to 45.1) | 44.6 (40.9 to 48.2) | 38.1 (34.3 to 41.9) | 46.7 (43.4 to 50.0) | 50.2 (46.2 to 54.1) | 53.3 (49.1 to 57.4) | 48.9 (41.3 to 56.6) |
| ULP | 2.2 (1.1 to 3.3) | **1.9 (0.9 to 3.0)** | 2.5 (1.3 to 3.8) | 3.0 (1.6 to 4.5) | 2.7 (1.6 to 3.8) | 6.0 (4.0 to 8.0) | 3.6 (2.0 to 5.3) | 5.7 (3.6 to 8.7) |
| Roll your own | 15.0 (12.4 to 17.7) | 19.7 (16.7 to 22.6) | 17.6 (14.7 to 20.4) | 20.4 (17.2 to 23.6) | 18.6 (16.0 to 21.2) | 18.0 (14.9 to 21.2) | 16.4 (13.3 to 19.6) | 19.5 (14.2 to 26.2) |
| Other/DNA | 3.4 (1.9 to 4.9) | 2.7 (1.4 to 4.1) | 1.2 (0.3 to 2.0) | 2.7 (1.3 to 4.2) | 2.7 (1.5 to 3.9) | 1.5 (0.4 to 2.6) | 3.3 (1.6 to 5.0) | 4.6 (2.7 to 7.8) |

^1^Confidence intervals calculated from simple random sample standard error and deft calculated using SPSS complex samples for 2008 data

^2^Confidence intervals from SPSS complex samples

Green shading – significant decrease from 2001

Yellow shading – significant increase from 2001

Bold text indicates low ULP rates in the middle of the decade
